# Calculation of the ovule number in the genus *Salix*: A method for taxa differentiation

**DOI:** 10.1002/aps3.11450

**Published:** 2021-12-08

**Authors:** Аlexander M. Marchenko, Yulia A. Kuzovkina

**Affiliations:** ^1^ Russian Park of Water Gardens Moscow Russia; ^2^ Department of Plant Sciences University of Connecticut Storrs Connecticut USA

**Keywords:** hybrid, identification, ovary, Salicaceae, *Salix* ×*bebbii*, *Salix fragilis*, taxonomy, willow

## Abstract

**Premise:**

Recent investigations have demonstrated that the number of ovules present in the ovaries of the willow flower (*Salix* spp., Salicaceae) can be used to confirm the identification of species and hybrids. We present the method to calculate the ovule number, along with examples demonstrating its use for both species and hybrid verification.

**Methods and Results:**

The best time to obtain a reliable ovule count is at the beginning of anthesis before numerous hairs develop in the ovary or after seed dispersal when the funiculi on the placenta can be counted. The ovules in all of the ovaries of one catkin should be counted, and the ovule index is recorded as their minimum–maximum range. The ovule number of a hybrid is the statistical mean of the ovule number of its parents.

**Conclusions:**

Ovule quantification is a useful tool that, in conjunction with traditional morphological and modern molecular techniques, presents additional evidence to support taxonomic decisions. The ovule number can also assist in species identification, classification, and in hybridization studies to verify the parentage of willow hybrids.

The genus *Salix* L. (Salicaceae), comprising approximately 450 species of woody plants (Argus, 2010), is one of the most difficult entities among woody plants for identification. Traditionally, willow identification is based on morphological characteristics, which can be used to distinguish many taxa within the genus. However, the high degree of variability within *Salix* limits the diagnostic value of many characters.

Surprisingly, little attention was initially given to the ovule number in numerous taxonomic works related to *Salix*, even though species descriptions included very thorough depictions of other morphological characters. Roxburgh (1795) was the first to mention the precise number of seeds (four) in the Indian species *S. tetrasperma* Roxb. while describing this new species. However, the ovule number was omitted in later monographs on *Salix*, including the influential mid‐20th century taxonomic treatments by Skvortsov (1968, 1999), Argus (1957, 1964, 1973, 1986), Rechinger (1964), Dorn (1976), Meikle (1984), and others. The first ovule counts were provided by Chmelař and Meusel (1976) and Chmelař (1977), who presented the ovule number for European *Salix* species. This was followed by Argus (1986, 2010) and Valyagina‐Malutina (2004), who reported the ovule number for North American and European species of *Salix*. Argus (1986) stated that “ovule number is potentially of taxonomic importance… more observations need to be made of this characteristic.”

## Morphology of the pistillate flowers

**Figure 1:**
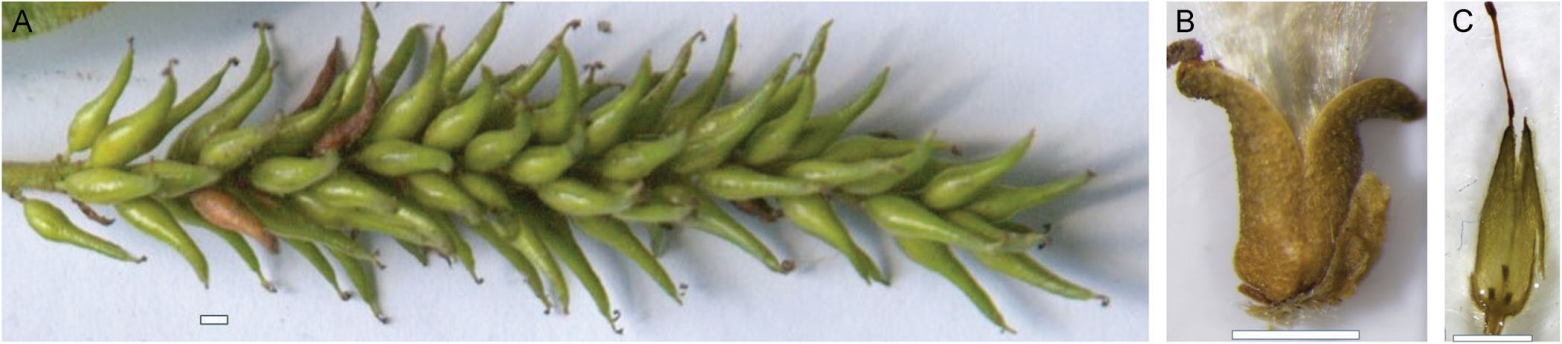
(A) A willow catkin with maturing capsules. (B) A ripe capsule with the two valves separated along the suture. (C) Three funiculi in the valve of the ovary after seed dispersal. Scale bars = 1 mm

The willow flowers are arranged into catkins (Figure 1A). Pistillate flowers consist of a single pistil with an ovary, floral bract, and nectary (or nectaries). The willow fruit is a capsule, formed by the fusion of two carpels. When ripe, the two capsule valves separate along the suture (the central vascular bundles of the carpels), exposing the seeds, but remain attached at the base (Figure 1B). Most open valves are curved or spiraled back.

The ovules are located on the placenta, which is positioned in the middle of the lower section of each valve. Following fertilization, the ovules develop into seeds. Capsule dehiscence and seed shedding typically occur 3–8 weeks after pollination. The matured fruits do not produce the maximum number of seeds, i.e., not all ovules develop into seeds. The natural seed set in *Salix* is generally less than 50% (even <20% in *S. alba*), which could be associated with pollen limitation or pre‐dispersal seed predation (Karrenberg et al., 2002). Because not all ovules develop into seeds, the number of seeds in a ripe capsule is not an accurate assessment of the number of ovules; instead, their funiculi should be used for counts (Figure 1C). Funiculi develop even if an ovule has not been fertilized, and consequently, no seed has developed.

## Recent studies

In our previous research, we have extensively explored the taxonomic importance of the ovule number in the genus *Salix* to confirm its biological and practical significance. Marchenko (2019) advanced the ovule number methodology to propose that the percentage of valves in a catkin with different possible numbers of ovules as well as the minimum and maximum number of ovules per ovary maximize the power of resolution and precision of genotype recognition. These detailed calculations facilitate a more robust methodology, allowing for differentiation between species, forms, and varieties.

Marchenko (2019) validated Chmelař's (1977) hypothesis that the ovule number of a hybrid is the statistical mean of the ovule number of its parents. The heritability of the ovule number in hybrid taxa was robustly tested using 30 cold‐hardy ornamental hybrid willows with known genetic background, developed in Russia in the 1960s (Marchenko, 2019). The ovule number data from the parents of these hybrids were used to calculate the predicted values for the ovule numbers, which were then compared to the actual ovule numbers in these hybrids; the results confirmed the accuracy of the hypothesis. Marchenko (2019) also enhanced the resolution power of Chmelař's formula for the identification of hybrids. Chmelař used only the number of ovules per valve when calculating the hybrid formula, whereas Marchenko used two quantifiers, the minimum and maximum number of ovules (i.e., the ovule indices), which more accurately represents the range of ovules in a hybrid. Marchenko further proposed that the percentage of valves with a different number of ovules has a diagnostic value, as these values differ between different genotypes.

We also used the quantification of ovules to confirm the hybrid origin of some *Salix* species in another study (Marchenko and Kuzovkina, 2021), wherein we verified the parentage of a few questionable taxa present in North America. This study confirmed the usefulness of the ovule number for the confirmation of the putative parents of the hybrids.

Because no detailed description of the procedure was ever provided, here we describe the method for calculating the ovule number, which can be used for identification of species and verification of the parentage of willow hybrids.

## METHODS AND RESULTS

**Figure 2:**
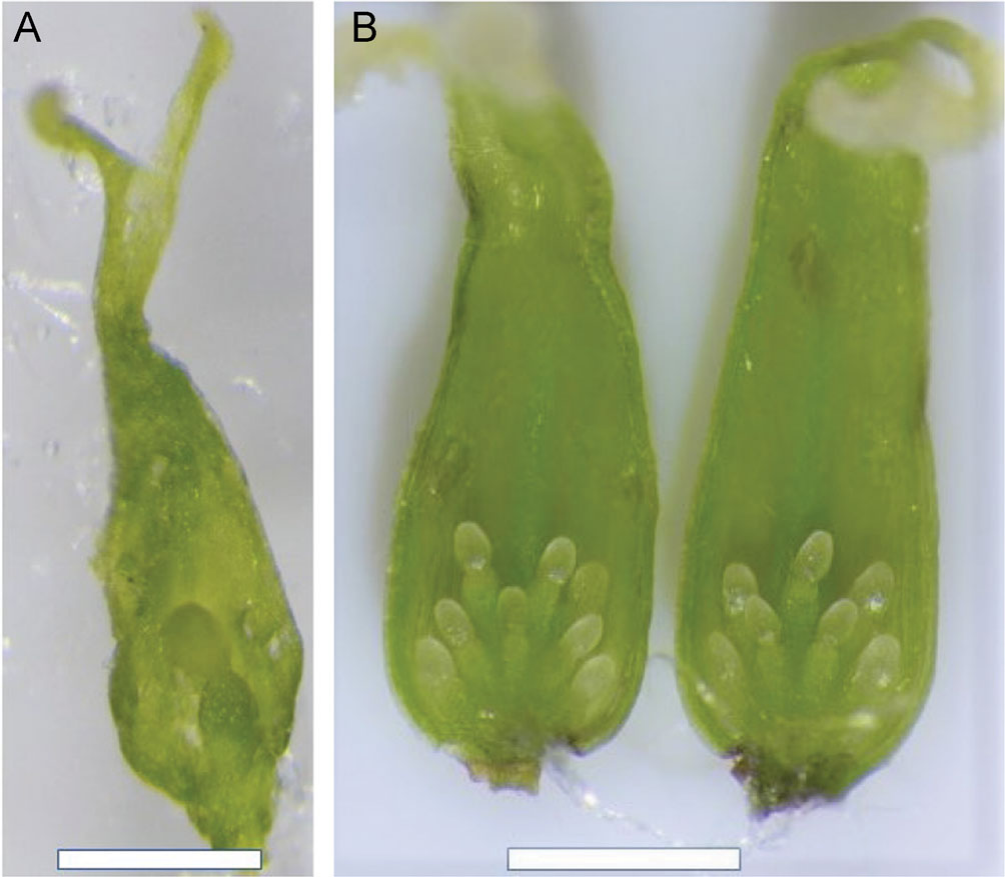
Detached ovary valves showing the variation in ovule numbers per ovary within *Salix* species. (A) *Salix eleagnos* has the lowest number, with only one ovule per valve. (B) Sixteen ovules on the two valves of the ovary of the hybrid *Salix* ‘Oranzhevaya Tolstostvol'naya’. Scale bars = 1 mm

The number of ovules per ovary in *Salix* ranges between two and 40 (Figure 2). In *Salix*, each species has a specific range of ovule numbers, which is called the ovule index. The data on the ovule indices for a large number of *Salix* species are presented in Table 1. This information can be used to assist in taxonomic studies and to calculate the *predicted ovule index* for hybrids described below.

**Table 1:** The ovule indices for various species of *Salix*, as reported by authors discussed in this study

Subgenus	Section^a^	Species	Ovule index (minimum–maximum number of ovules per ovary)			
			Argus, 2010	Chmelař, 1977	Valyagina‐Malutina, 2004	Marchenko, 2019
Protitea	Floridanae	*S. floridana*	4–4	—	—	—
	Tetraspermae	*S. tetrasperma*	—	4–4	—	4–4
	Humboldtianae	*S. caroliniana*	12–16	—	—	—
		*S. longipes* b	—	12–12	—	—
		*S. nigra*	12–16	—	—	—
		*S. bonplandiana*	8–18	12–16	—	—
		*S. gooddingii*	12–18	—	—	—
		*S. amygdaloides*	16–18	—	—	—
		*S. laevigata*	12–24	—	—	—
		*S. humboldtiana*	—	20–24	—	—
		*S. mucronata*		—	—	12–12
Pleuradenia		*S. cardiophylla*	—	4–4	—	4–4
		*S. arbutifolia*	—	4–4	—	4–4
Salix	Subalbae	*S. babylonica*	2–4	4–4	—	2–5
		*S. pierotii*	—	4–4	—	4–4
	Salix	*S. fragilis*	—	6–8	6–6	6–6
		*S. alba*	8–9	12–16	6–10	10–24
		*S. micans*	—	14–16	—	—
	Salicaster	*S. pseudopentandra*	—	12–12	—	—
		*S. serissima*	12–16	—	—	15–17
		*S. pentandra*	18–22	22–24	16–24	18–24
		*S. lucida*	18–24	—	—	—
		*S. paraplesia*	—	22–24	—	—
		*S. lasiandra*	16–30	—	—	—
	Maccallianae	*S. maccalliana*	12–16	—	—	—
	Triandrae	*S. triandra*	30–36	28–30	30–40	—
Longifoliae		*S. melanopsis*	13–22	—	—	—
		*S. taxifolia*	16–26	—	—	—
		*S. exigua*	12–30	28–32	—	—
		*S. columbiana*	18–30	—	—	—
		*S. interior*	16–36	24–28	—	21–34
		*S. thurberi*	16–36	—	—	—
		*S. sessilifolia*	24–36	—	—	—
Chamaetia	Chamaetia	*S. nivalis*	8–10	—	—	—
		*S. reticulata*	8–18	10–18	6–10	11–16
		*S. vestita*	13–15	—	—	—
	Setchellianae	*S. setchelliana*	16–23	—	—	—
	Herbella	*S. serpyllifolia*	—	—	—	6–10
		*S. nummularia*	8–10	—	—	—
		*S. retusa*	—	12–16	—	—
		*S. rotundifolia*	7–17	—	—	—
		*S. polaris*	12–17	—	10–12	—
		*S. herbacea*	11–18	—	10–12	—
	Myrtosalix	*S. alpina*	—	6–8	—	—
		*S. uva‐ursi*	4–9	—	—	—
		*S. fuscescens* c	8–12	—	—	—
		*S. phlebophylla*	12–12	—	—	—
		*S. myrsinites*	—	—	10–12	—
		*S. arctophila*	8–16	—	—	—
		*S. saxatilis*	—	—	—	11–16
		*S. chamissonis*	12–18	—	—	—
	Ovalifoliae	*S. stolonifera*	12–13	—	—	—
		*S. ovalifolia*	10–15	—	—	—
		*S. jejuna*	9–18	—	—	—
	Diplodictyae	*S. cascadensis*	6–10	—	—	—
		*S. petrophila*	6–12	—	—	—
		*S. arctica*	12–15	—	16–18	—
		*S. sphenophylla*	10–18	—	—	—
	Myrtilloides	*S. pedicellaris*	4–6	—	—	—
		*S. myrtilloides*	—	6–6	6–6	—
		*S. chlorolepis*	8–10	—	—	—
		*S. raupii*	12–12	—	—	—
		*S. athabascensis*	6–14	—	—	—
	Glaucae	*S. brachycarpa*	2–10	—	—	—
		*S. reptans*	—	—	6–10	—
		*S. niphoclada*	8–20	—	—	—
		*S. glauca*	6–22	12–16	10–12	—
		*S. pyrenaica*	—	—	—	11–17
		*S. nakamurana*	—	—	—	11–17
Vetrix	Hastatae	*S. arizonica*	8–12	—	—	—
		*S. myrtillifolia*	10–14	—	—	—
		*S. myricoides*	12–14	—	—	11–12
		*S. monticola*	11–15	—	—	—
		*S. wolfii*	8–16	—	—	—
		*S. eastwoodiae*	12–16	—	—	—
		*S. orestera*	15–16	—	—	—
		*S. pyrolifolia*	—	—	10–12	16–16
		*S. boothii*	10–18	—	—	—
		*S. pseudomyrsinites*	11–18	—	—	—
		*S. ballii*	12–18	—	—	—
		*S. pseudomonticola*	18–18	—	—	—
		*S. pyrifolia*	10–19	—	—	—
		*S. farriae*	12–19	—	—	—
		*S. hastata*	12–22	—	16–18	—
		*S. cordata*	11–24	10–18	—	15–18
		*S. barclayi*	18–24	—	—	—
		*S. commutata*	10–28	—	—	—
	Glabrella	*S. glabra*	—	6–8	—	—
	Cordatae	*S. eriocephala*	12–16	—	—	11–18
		*S. famelica*	12–18	—	—	—
		*S. turnorii*	14–18	—	—	—
		*S. ligulifolia*	12–21	—	—	—
		*S. monochroma*	18–22	—	—	—
		*S. prolixa*	12–22	—	—	—
		*S. lutea*	12–24	—	—	—
	Nigricantes	*S. myrsinifolia*	12–14	—	12–16	—
	Cinerella	*S. humilis*	6–12	—	—	—
		*S. aurita*	10–12	—	12–16	—
		*S. cinerea*	12–12	—	12–16	—
		*S. atrocinerea*	12–12	—	—	—
		*S. caprea*	12–14	12–14	16–18	—
		*S. discolor*	6–16	—	—	—
		*S. scouleriana*	10–18	—	—	—
		*S. hookeriana*	12–20	—	—	—
	Fulvae	*S. bebbiana*	6–16	—	—	—
	Phylicifoliae	*S. basaltica*	—	12–14	—	—
		*S. bicolor*	—	12–14	—	—
		*S. hegetschweilerii* d	—	12–14	—	—
		*S. phylicifolia*	—	12–14	—	—
		*S. planifolia*	11–16	—	—	—
		*S. pulchra*	12–16	—	10–12	—
		*S. tyrrellii*	12–16	—	—	—
		*S. drummondiana*	6–17	—	—	—
		*S. pellita*	10–18	—	—	—
	Arbuscella	*S. arbuscula*	—	—	10–12	—
		*S. arbusculoides*	16–18	—	—	—
	Candidae	*S. candida*	12–18	—	—	—
	Lanatae	*S. lanata*	—	—	16–18	14–19
		*S. calcicola*	13–20	—	—	—
		*S. tweedyi*	18–30	—	—	—
		*S. richardsonii*	22–37	—	—	—
	Villosae	*S. lapponum*	—	10–10	12–16	14–18
		*S. silicicola*	12–14	—	—	—
		*S. alaxensis*	14–18	—	—	10–16
		*S. barrattiana*	16–21	—	—	—
	Viminella	*S. schwerinii*	—	—	—	12–12
		*S. udensis*	—	—	—	12–16
		*S. dasyclados*	—	—	12–16	—
		*S. viminalis*	12–18	16–16	16–18	—
	Subviminales	*S. gracilistyla*	—	—	—	4–6
	Canae	*S. elaeagnos*	2–2	2–2	—	2–2
	Argyrocarpae	*S. argyrocarpa*	12–13	—	—	—
	Geyerianae	*S. petiolaris*	6–12	—	—	—
		*S. geyeriana*	6–12	—	—	—
		*S. lemmonii*	12–12	—	—	—
	Mexicanae	*S. irrorata*	9–12	—	—	—
		*S. tracyi*	12–12	—	—	—
		*S. lasiolepis*	10–18	—	—	—
	Griseae	*S. sericea*	6–6	—	—	—
	Sitchenses	*S. breweri*	4–12	—	—	—
		*S. jepsonii*	13–18	—	—	—
		*S. delnortensis*	14–18	—	—	—
		*S. sitchensis*	14–20	—	—	—
	Daphnella	*S. daphnoides*	4–6	6–6	—	6–6
		*S. kangensis*	—	—	—	6–6
		*S. rorida*	—	—	—	6–6
		*S. acutifolia*	—	—	6–6	—
	Incubaceae	*S. rosmarinifolia*	—	—	6–6	—
		*S. repens*	—	6–6	—	6–6
	Helix	*S. purpurea*	6–6	6–6	—	6–6
		*S. integra*	—	6–6	—	6–6
		*S. kochiana*	—	—	—	6–6
		*S. cаesia*	—	6–6	—	6–6
		*S. ledebouriana*	—	—	—	6–6
		*S. miyabeana*	—	—	—	6–6
		*S. vinogradovii*	—	—	6–6	6–6
		*S. elbursensis*	—	6–6	—	—
		*S. caspica*	—	6–6	—	—

^a^ The classification is based on Argus (2010).

^b^ *Salix longipes* is a synonym of *S. caroliniana* (Argus, 2010).

^c^ *Salix fuscescens* belongs to the section *Myrtilloides* according to Skvortsov (1968).

^d^ *Salix hegetschweilerii* is a synonym of *S. phylicifolia* according to Skvortsov (1968).

The ovule index can be used in combination with traditional morphological characteristics to confirm the identification of a specimen in question. This method is very useful for distinguishing morphologically similar species with non‐overlapping ovule indices.

### Specimen preparation

**Figure 3:**
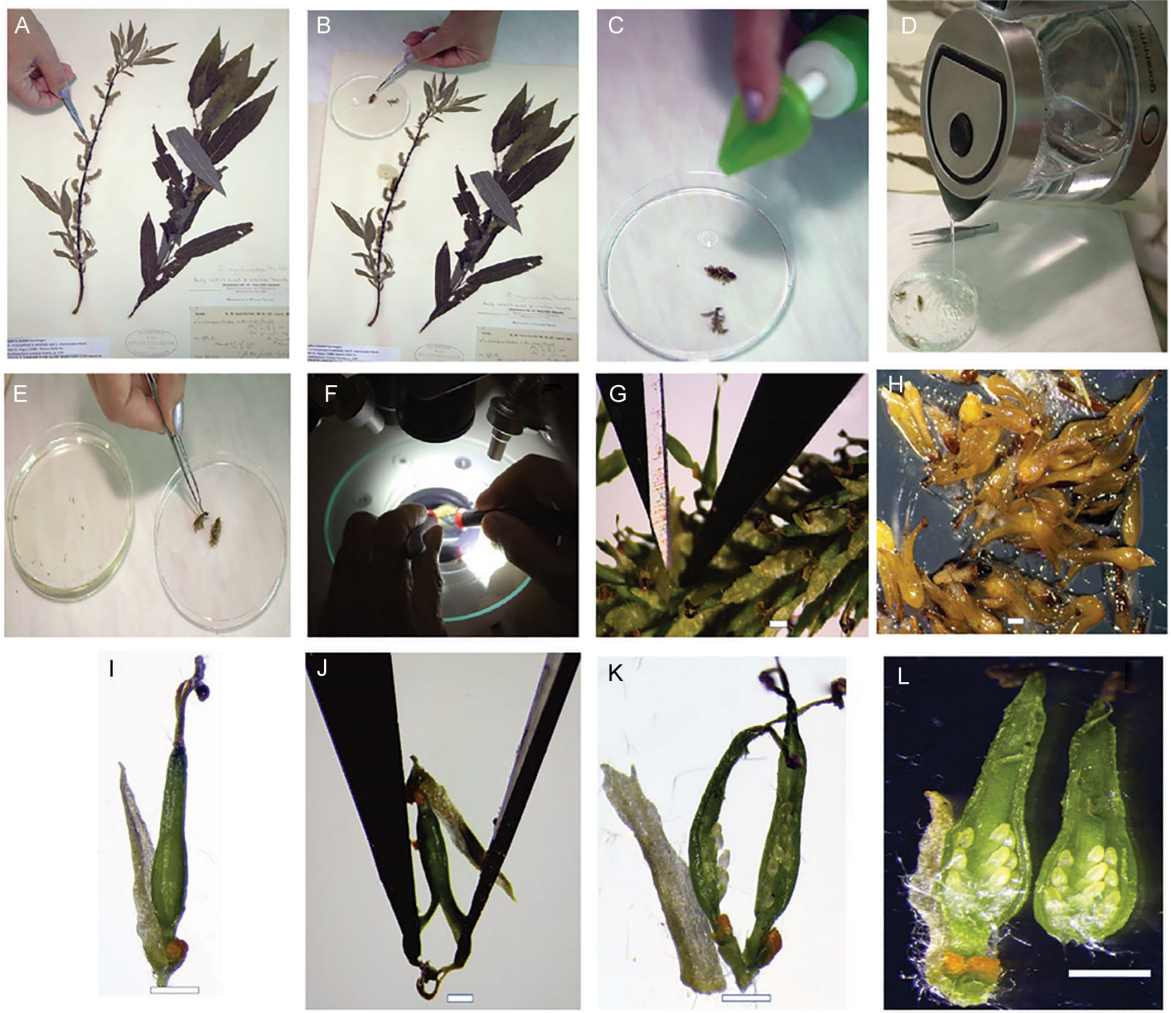
Step‐by‐step demonstration of the method to calculate the ovule number in immature ovaries using herbarium specimens. (A) Using forceps, remove a catkin from the middle of the branchlet. (B) If there is glue adhering to the catkin, soak it in a drop of water for 2–3 min. (C) Place the catkin in a Petri dish, and smear a drop of detergent near the catkin. (D) To soften the tissues, pour boiling water into a Petri dish and soak the catkin for 15–30 min. (E) Move the catkin into a dry Petri dish and (F) place under the microscope. (G) Using two X‐Acto knives, detach all ovaries from the catkin rachis. (H) Place all detached ovaries together and discard the rest of the catkin. (I) A detached ovary with a bract, nectary, and stipe. (J) Using two X‐Acto knives, open the ovary by separating the two valves along the central veins of the carpels. (K) Two valves of the ovary attached at the base. (L) Two detached valves of the ovary with the exposed ovules prepared for counting. Scale bars = 1 mm

To determine the ovule index for a specimen, one catkin from the middle part of the crown should be examined and the ovules per valve in all ovaries of the catkin should be counted (Appendix 1). The number of ovules can be quantified in either female or androgynous plants (often androgynous plants occur among hybrids) using live or herbarium specimens. When using herbarium specimens, the catkins should be covered with boiling water with a small amount of detergent and soaked for 15–30 min to soften the tissues (Figure 3D).

**Figure 4:**
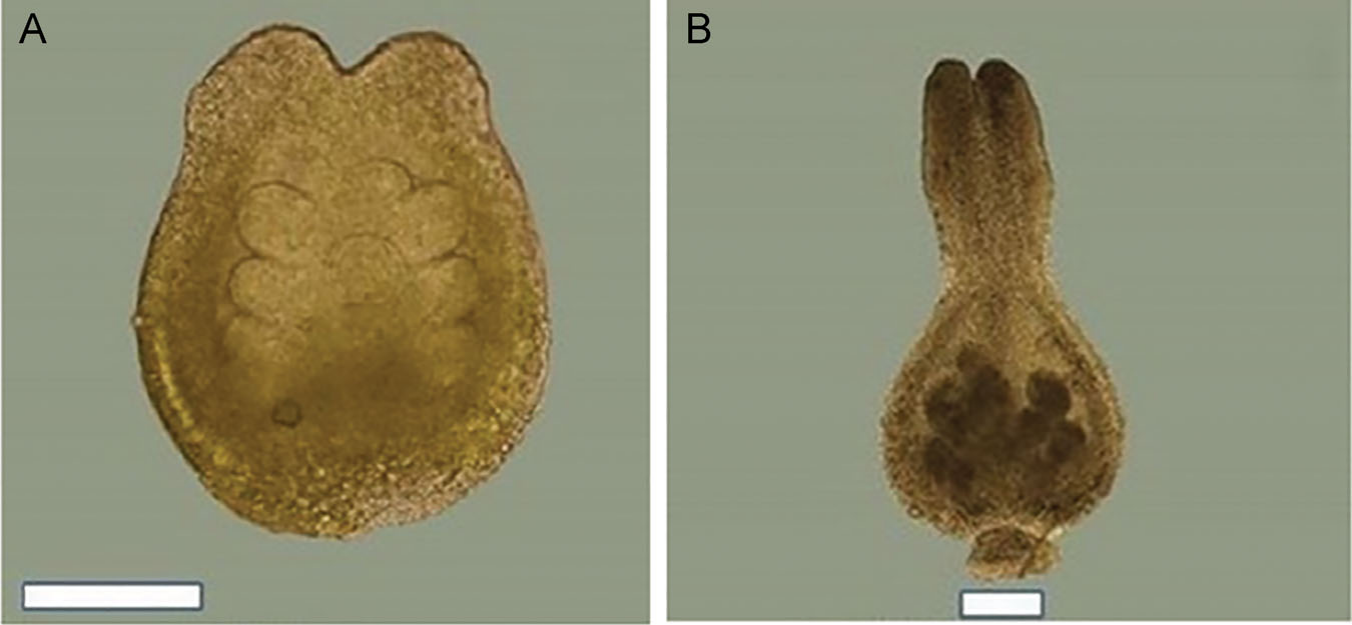
A valve of the ovary of *Salix schwerenii*. The immature ovules within a developing ovary inside the lateral bud are visible at the end of summer (A) and in winter (B). Scale bars = 1 mm

There are a few phenological phases during which it is possible to make reliable ovule counts. The best time is at the beginning of anthesis, just before fertilization takes place (Figure 2); after fertilization, numerous hairs develop in the ovary, making it more difficult to count the ovules. The forcible opening of the immature ovary should be performed along the central vein of the carpel, which prevents damage to the ovules and funiculi. The second‐best time is after seed dispersal, when the funiculi on the placenta from both undeveloped ovules and developed seeds can be counted. An optical microscope should be used for ovule and funiculi magnification during counts (for example, we used a Nikon SMZ800N stereomicroscope [Nikon, Tokyo, Japan]). If there is an urgent need, the immature ovules can also be calculated in the early stages of their development inside the generative buds at the end of summer and throughout winter using high magnification (Figure 4).

### Ovule quantification

The ovule index is recorded as the minimum–maximum range of ovules per ovary in a catkin (for example, *n* = 10–12). For each specimen, the percentages of valves with different numbers of ovules should also be determined (Appendix 2).

For F_1_ hybrid verification, when the ovule index is obtained by counting the ovules in a studied specimen, it is called the *calculated ovule index*. When it is obtained by calculating the statistical mean of the ovule number for two parents, it is called the *predicted ovule index*.

If the calculated ovule index coincides with the predicted ovule index for an unconfirmed hybrid whose parent species have been proposed based on morphological characters, then this specimen is confirmed as a hybrid between these parent species. If the indices do not match, a different parent couple should be suggested.

When identifying a male parent of a hybrid, the number of ovules known for female representatives of the species is used in the calculation, as male plants pass on the genetic information regarding the number of ovules characteristic of the taxon.

For hybrids, the ovule data can be presented in a convenient table format (Appendix 3) that also includes the ovule numbers for the parent species. The ovule values for a hybrid will occupy the intermediate positions between the parents.

### Application of the method

The following two examples demonstrate the proposed technique for the identification of willow species (Example 1) and for hybrid verification (Example 2).

**Figure 5:**
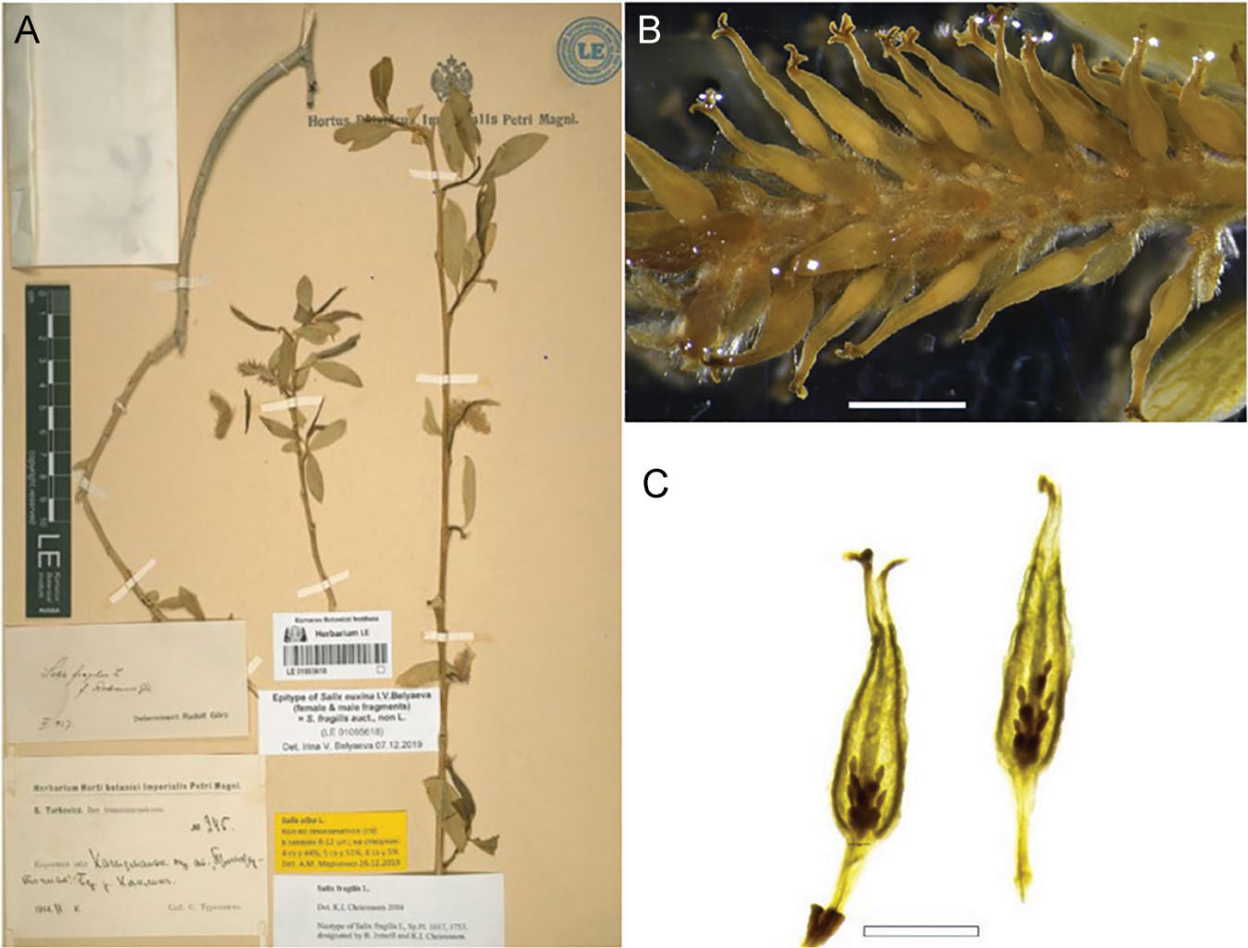
A herbarium specimen of *Salix fragilis* (LE 01065618) and its flower details. (A) Herbarium specimen. (B) The catkin of LE 01065618 with 38 ovaries used for the ovule count. (C) A dissected ovary of LE 01065618 with 10 ovules (five ovules in each valve). Scale bars = 1 mm

**Example 1:** We analyzed the specimen LE 01065618 identified as *S. fragilis* L. by Turkevicz in 1914 from the Herbarium of the Komarov Botanical Institute of the Russian Academy of Sciences (Figure 5). *Salix fragilis* is a Eurasian willow tree species. The specimen LE 01065618 was identified and cited as a pure *S. fragilis* by various authors (Skvortsov, 1973; Skvortsov and Edmonson, 1982; Christensen and Jonsell, 2005; Belyaeva, 2009). We analyzed all ovaries of one catkin (*n* = 38) from this specimen and recorded the ovule number in each of the two valves in all ovaries. We documented 44% of valves with four ovules, 51% of valves with five ovules, and 5% of valves with six ovules. The ovule index, as the minimum–maximum number of ovules per ovary, was 8–12. As per previous investigations, all valves of *S. fragilis* have three ovules, resulting in an ovule index of 6–6 (Chmelař, 1977; Valyagina‐Malutina, 2004; Marchenko, 2019). The ovule index for the specimen LE 01065618 did not match the previously reported value. Thus, the ovule number did not confirm the identification of this specimen as *S. fragilis* and another identification was suggested. We proposed that based on the ovule index 8–12, in conjunction with other morphological characters of this specimen (for example, cataphylls with short trichomes), it is correctly identified as *S. alba* L.

*Salix fragilis* and *S. alba* both occur in the area where LE 01065618 was collected. These species are not easy to differentiate based on traditional morphological characteristics, but they are easily distinguishable by the ovule number.

**Figure 6:**
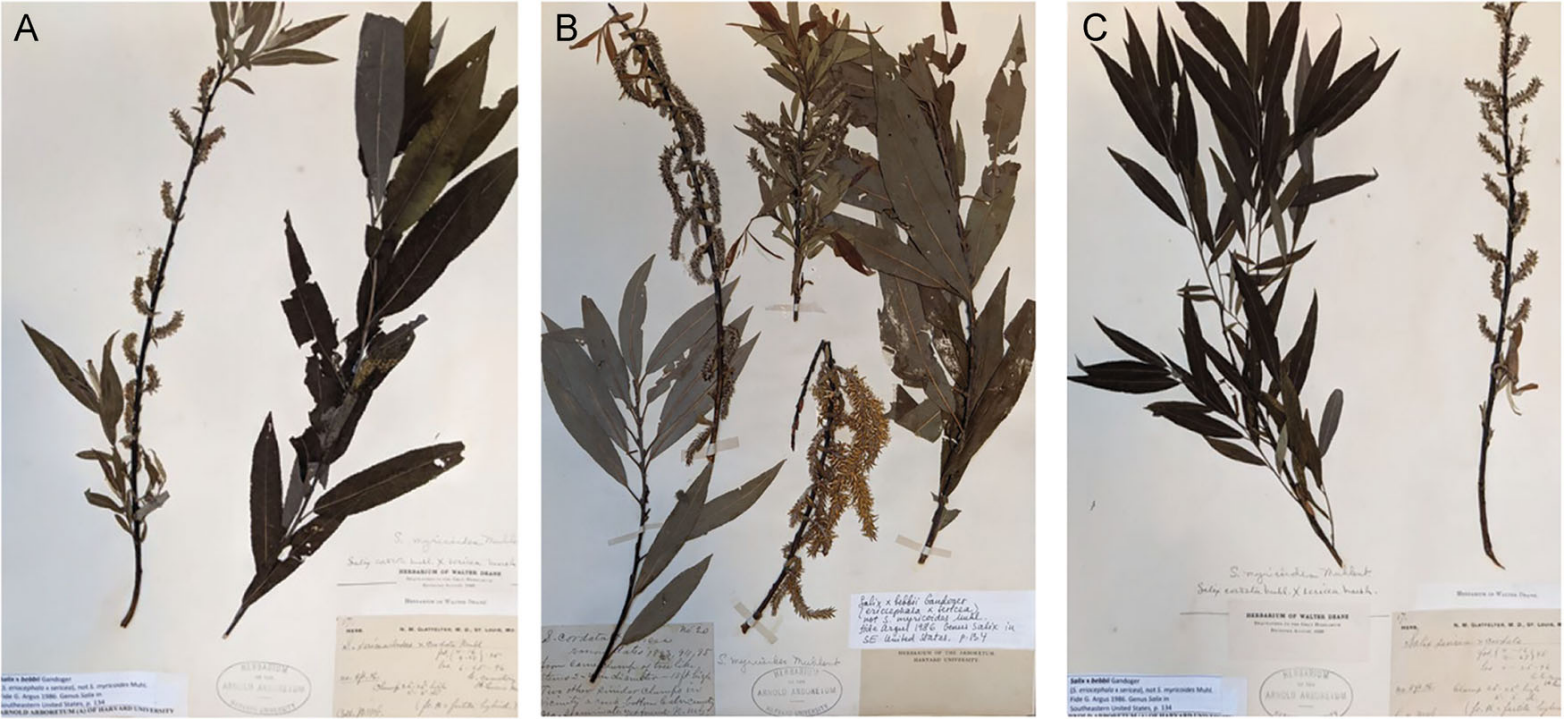
Herbarium specimens identified by G. Argus as *Salix* ×*bebbii*. (A) Specimen 1. (B) Specimen 2. (C) Specimen 3

**Example 2:** We analyzed three specimens from the Herbarium of the Arnold Arboretum, identified by G. Argus as *S*. ×*bebbii* Gand. *Salix* ×*bebbii* (*S. eriocephala* Michx. × *S. sericea* Marshall) is a North American hybrid that is relatively common wherever the parent ranges overlap. These specimens were studied by Argus for the purpose of his taxonomic revision “The genus *Salix* (Salicaceae) in the southeastern United States” (Argus, 1986). We noticed that these three specimens varied morphologically, although they were all identified as *S*. ×*bebbii* by Argus (Figure 6).

First, using the known ovule indices of the proposed parent species *S. eriocephala* (ovule index 12–16) and *S. sericea* (ovule index 6–6), we determined that the predicted ovule index for their hybrid offspring should be within the range of 9–11. We estimated this as follows:

the mean of the minimum ovule number of the parents: (12 + 6)/2 = 18/2 = 9;
the mean of the maximum ovule number of the parents: (16 + 6)/2 = 22/2 = 11.

Next, we counted the ovules in all ovaries of the catkin from each specimen (i.e., 56 ovaries in Specimen 1, 78 ovaries in Specimen 2, and 64 ovaries in Specimen 3) and determined the calculated ovule indices following the methodology outlined above.

Specimen 1: 9–11 (9: 40%, 10: 40%, 11: 20%)
Specimen 2: 9–13 (9: 25%, 10: 12.5%, 11: 50%, 13: 12.5%)
Specimen 3: 3–7 (3: 25%, 6: 25%, 7: 50%)

Thus, in the catkins of Specimens 1 and 3 we recorded three types of ovaries (with nine, 10, or 11 ovules per ovary in Specimen 1, and with three, six, or seven ovules per ovary in Specimen 3), while we recorded four types of ovaries in the catkin of Specimen 2 (with nine, 10, 11, or 13 ovules per ovary).

The calculated index for Specimen 1 coincided with the predicted index (9–11), confirming that this specimen was a hybrid between *S. eriocephala* and *S. sericea*. For Specimens 2 and 3, the calculated and predicted indices did not match, and therefore another identification should be suggested. Our analyses also indicated that Specimen 1 had intermediate morphological characters between both parents, as was expected from a hybrid specimen, while Specimens 2 and 3 were morphologically different from their proposed parents. Thus, the ovule number confirmed Argus's identification of Specimen 1, but did not support the identification of Specimens 2 and 3 as *Salix* ×*bebbii*.

## CONCLUSIONS

With the advent of molecular techniques, the ovule count provides another analytical tool for species identification in the genus *Salix*. Combined with traditional morphological and modern molecular techniques, the ovule number presents additional evidence to support taxonomic decisions and classification systems. The ovule number can also be used in hybridization studies to verify the parentage of willow hybrids. In some cases, the ovule number can detect hybridization and reveal hybrid taxa that were not obvious from morphological characters. Breeding programs can use this technique for the selection of individuals and certification of controlled crosses.

## AUTHOR CONTRIBUTIONS

A.M.M. created the technique and collected data; Y.A.K. wrote the first draft of the manuscript. Both authors reviewed, edited, and approved the final manuscript before submission and publication.

## Data Availability

The high‐resolution images of the herbarium specimens used in this study are available at the Center for Open Science's Open Science Framework (https://osf.io/kdqm5/).

## Appendix 1 Protocol for obtaining the ovule count using herbarium specimens

### Materials

1. A pair of forceps
2. Two Petri dishes
3. Small amount of detergent
4. Two X‐Acto knives
5. An optical microscope
6. Boiling tap water

### Procedure

Step 1: Place the specimen on a flat surface and use a pair of forceps to manually remove a catkin from the middle of the branchlet. If there is glue adhering to the catkin, soak it in a drop of tap water for 2–3 min.
Step 2: Place the catkin in a Petri dish; smear a drop of detergent near the catkin.
Step 3: To soften the tissues, pour a small amount of boiling tap water in the Petri dish and allow the catkin to soak for 15–30 min.
Step 4: After soaking, move the catkin into a dry Petri dish using forceps and place under the microscope.
Step 5: Using two X‐Acto knives, detach all ovaries from the catkin rachis.
Step 6: Place all detached ovaries together and discard the rest of the catkin.
Step 7: Using two X‐Acto knives, open the ovary by pulling the two valves along the central veins of the carpels to expose the ovules.
Step 8: Count the ovules in each valve of all ovaries and record the data.
Step 9: Calculate the percentage of valves with a specific number of ovules and the percentage of ovaries with a specific number of ovules in a catkin.
Step 10: Calculate the ovule index as the minimum–maximum range of ovules per ovary. For example, in one catkin of *Salix* ×*cottetii* there are 70 ovaries; each ovary should be opened and the ovules in each of the two valves should be counted. Data are recorded in the following format: 3/5 (8) – 1 (1%), in which the first two digits, written with the forward slash, indicate the number of ovules in each of the two valves; the number in parentheses indicates the number of ovules per ovary; the number following the dash represents the number of ovaries in the catkin with the given range of ovules; and the percentage given in parentheses represents the percentage of the ovaries in the catkin with this count. Thus, the record states: 3/5 (8) – 1 (1%); 4/5 (9) – 3 (5%); 5/5 (10) – 29 (41%); 5/6 (11) – 17 (25%); 6/6 (12) – 20 (28%), where the minimum number of ovules per ovary is eight and the maximum is 12, so the ovule index is 8–12.
Step 11: Summarize the data in table format.

## Appendix 2 The ovule data for 50 *Salix* taxa from Marchenko's collection. The presented data include the percentage of valves with different possible numbers of ovules and the ovule indices (the minimum and maximum number of ovules per ovary) in the studied specimens

**Table:**

Species	Percentage of valves with each ovule number											Ovule index
	1	2	3	4	5	6	7	8	9	10	11	
*S.* ‘Aegma Brno’					18	59	13	10				10–16
*S. alaxensis*				3	21	43	12	21				10–16
*S. ×boydii*						7	33	60				13–16
*S. cаesia*			100									6–6
*S. caprea* ‘Ogon’					4	83	13					11–13
*S. caprea* ‘Ogon’ × sp. indet.					14	73	10	3				10–14
*S. cardiophylla*		100										4–4
*S. cordata*							9	40	49	2		15–18
*S. daphnoides* var. *pomeranica*			100									6–6
*S. elaeagnos*	100											2–2
*S. eriocephala* ‘Russeliana’							6	13	81			16–18
*S. eriocephala* var. *watsonii*					3	6	22	50	19			11–18
*S. fargesii*		20	76	4								4–7
*S. glauca* var. *callicarpaea*						11	23	36	30			14–18
*S. integra* ‘Hakuro Nishiki’			100									6–6
*S. integra* ‘Pendula’			100									6–6
*S. integra* × *S. kochiana*			100									6–6
*S. interior* ^a^												
*S. kangensis*			100									6–6
*S. kochiana*			100									6–6
*S. lanata*						8	8	20	58	6		14–19
*S. lanata* (from Latvia)							22	51	27			14–18
*S. lapponum* (no. 1)							26	74				14–16
*S. lapponum* (no. 2)						5	20	50	25			12–18
*S. lapponum* (large specimen)								63	37			16–18
*S. lapponum* × *S. purpurea*				8	81	11						9–11
*S. ledebouriana* f. *kuraica*		20	80									4–6
*S. ledebouriana* var. *pyramidalis*			100									6–6
*S. leucopithecia*					38	62						11–12
*S. magnifica*							23	34	34	6	2	14–20
*S. miyabeana*			100									6–6
*S. nakamurana*		25	52	23								4–7
*S. pentandra* ^a^												
*S. pierotii*		100										4–4
*S. purpurea* ‘Pendula’			100									6–6
*S. purpurea* ‘Nana’	10	21	55									2–6
*S. pyrenaica*					8	40	21	23	8			11–17
*S. pyrolifolia*								100				16–16
*S. repens* subsp. *arenaria* (no. 1)			100									6–6
*S. repens* subsp. *arenaria* (no. 2)			95	5								6–8
*S. repens* (no. 1)			10	18	72							7–10
*S. repens* (no. 2)			7	16	70	3						7–11
*S. reptans*					8	30	21	33	8			11–17
*S. reticulata* (from Scotland)				3	3	91	3					11–13
*S. reticulata* (Kola Peninsula)					20	48	22	9				11–16
*S. rorida* ‘Pendula’			95	5								6–7
*S. saxatilis* (no. 1)					6	72	22					11–13
*S. saxatilis* (no. 2)					3	42	35	11	9			12–16
*S. serpyllifolia*			57	35	8							6–10
*S. vinogradovi*			100									6–6

^a^ These specimens had higher numbers of ovules per valve. *Salix interior*: 10 ovules (3%), 11 ovules (6%), 12 ovules (14%), 13 ovules (27%), 14 ovules (22%), 15 ovules (6%), 16 ovules (14%), 17 ovules (8%) (ovule index 21–34); *Salix pentandra*: 9 ovules (32%), 10 ovules (48%), 11 ovules (18%), 13 ovules (1%), 14 ovules (1%) (ovule index 18–24).

## Appendix 3 The percentages of ovaries in a catkin with a specific number of ovules and the ovule index (the minimum to maximum number of ovules per ovary) in *Salix* ×*cottetii*, *S. retusa*, and *S. myrsinifolia* (Marchenko and Kuzovkina, 2021)

**Table:**

Specimens	Percentage of ovaries with each ovule number^a^									Ovule index
	6	7	8	9	10	11	12	13	15	
*S.* ×*cottetii*			1	5	41	25	28			8–12
*S. retusa*	6	21	46	27						6–9
*S. myrsinifolia*					5	14	72	4	5	10–15

^a^All values represent the percentage of ovaries with different number of ovules in the catkin; empty cells represent the absence of ovaries with a specific number of ovules in the catkin.
